# Supplementary material for: HIVprotI: an integrated web based platform for prediction and design of HIV proteins inhibitors
Source: J Cheminform. 2018 Mar 9;10:12. doi: 10.1186/s13321-018-0266-y (PMC5845081; doi:10.1186/s13321-018-0266-y)
Supplement: Supplementary file 2 — Additional file 2. Source code of HIVProtI web server. [file 13321_2018_266_MOESM2_ESM.zip › HIVprotI_Source-code/hivprotI/weka-3-6-6/doc/weka/clusterers/Clusterer.html]

Clusterer


---


|  |  |  |
| --- | --- | --- |
| **Overview** \| **Package** \| **Class** \| **Tree** \| **Deprecated** \| **Index** \| **Help** \| **Weka's home** | |  |
| **PREV CLASS**   **NEXT CLASS** | **FRAMES**    **NO FRAMES**     **All Classes** |  |
| SUMMARY: NESTED \| FIELD \| CONSTR \| METHOD | DETAIL: FIELD \| CONSTR \| METHOD |  |


---


## weka.clusterers Interface Clusterer

**All Known Subinterfaces:**: DensityBasedClusterer

**All Known Implementing Classes:**: AbstractClusterer, AbstractDensityBasedClusterer, CLOPE, Cobweb, DBScan, EM, FarthestFirst, FilteredClusterer, HierarchicalClusterer, MakeDensityBasedClusterer, OPTICS, RandomizableClusterer, RandomizableDensityBasedClusterer, RandomizableSingleClustererEnhancer, sIB, SimpleKMeans, SingleClustererEnhancer, XMeans

---

``` public interface Clusterer ```

Interface for clusterers. Clients will typically extend either
AbstractClusterer or AbstractDensityBasedClusterer.

**Author:**
:   Mark Hall (mhall@cs.waikato.ac.nz)

---

| **Method Summary** | |
| --- | --- |
| `void` | `buildClusterer(Instances data)`             Generates a clusterer. |
| `int` | `clusterInstance(Instance instance)`             Classifies a given instance. |
| `double[]` | `distributionForInstance(Instance instance)`             Predicts the cluster memberships for a given instance. |
| `Capabilities` | `getCapabilities()`             Returns the Capabilities of this clusterer. |
| `int` | `numberOfClusters()`             Returns the number of clusters. |

**Method Detail**

### buildClusterer

```
void buildClusterer(Instances data)
                    throws java.lang.Exception
```

:   Generates a clusterer. Has to initialize all fields of the clusterer
    that are not being set via options.

    :   **Parameters:**: `data` - set of instances serving as training data **Throws:**: `java.lang.Exception` - if the clusterer has not been generated successfully

---


### clusterInstance

```
int clusterInstance(Instance instance)
                    throws java.lang.Exception
```

:   Classifies a given instance. Either this or distributionForInstance()
    needs to be implemented by subclasses.

    :   **Parameters:**: `instance` - the instance to be assigned to a cluster **Returns:**: the number of the assigned cluster as an integer **Throws:**: `java.lang.Exception` - if instance could not be clustered successfully

---


### distributionForInstance

```
double[] distributionForInstance(Instance instance)
                                 throws java.lang.Exception
```

:   Predicts the cluster memberships for a given instance. Either
    this or clusterInstance() needs to be implemented by subclasses.

    :   **Parameters:**: `instance` - the instance to be assigned a cluster. **Returns:**: an array containing the estimated membership probabilities of the test instance in each cluster (this should sum to at most 1) **Throws:**: `java.lang.Exception` - if distribution could not be computed successfully

---


### numberOfClusters

```
int numberOfClusters()
                     throws java.lang.Exception
```

:   Returns the number of clusters.

    :   **Returns:**: the number of clusters generated for a training dataset. **Throws:**: `java.lang.Exception` - if number of clusters could not be returned successfully

---


### getCapabilities

```
Capabilities getCapabilities()
```

:   Returns the Capabilities of this clusterer. Derived classifiers have to
    override this method to enable capabilities.

    :   **Returns:**: the capabilities of this object **See Also:**: `Capabilities`


---


|  |  |  |
| --- | --- | --- |
| **Overview** \| **Package** \| **Class** \| **Tree** \| **Deprecated** \| **Index** \| **Help** \| **Weka's home** | |  |
| **PREV CLASS**   **NEXT CLASS** | **FRAMES**    **NO FRAMES**     **All Classes** |  |
| SUMMARY: NESTED \| FIELD \| CONSTR \| METHOD | DETAIL: FIELD \| CONSTR \| METHOD |  |


---
